# Phyto-fabrication of silver nanoparticles and their catalytic dye degradation and antifungal efficacy

**DOI:** 10.3389/fchem.2022.994721

**Published:** 2022-09-26

**Authors:** Chanda Kumari Githala, Shani Raj, Anita Dhaka, Suresh Chand Mali, Rohini Trivedi

**Affiliations:** Laboratory of Plant Pathology, Department of Botany, Mohanlal Sukhadia University Udaipur, Rajasthan, India

**Keywords:** silver nanoparticles, green synthesis, Plantago ovata, characterization, dye degradation, antifungal activity

## Abstract

The biogenic synthesis of silver nanoparticles (AgNPs) and their potent application against dye degradation and phytopathogens are attracting many scientists to nanotechnology. An attempt was made to synthesize silver nanoparticles using *Plantago ovata* leaf extract and test their effectiveness in removing organic dyes and antifungal activity. In the present study, stable AgNPs were synthesized from 0.1 mM AgNO_3_ and authenticated by observing the color change from yellow to red-brown, which was confirmed with wavelength UV-Vis spectrophotometer detection. The crystalline nature of the particles was characterized by x-ray diffraction (XRD) patterns. Furthermore, the AgNPs were characterized by high-resolution transmission electron microscope and scanning electron microscope investigations. Atomic force microscopy (AFM) and Raman spectra were also used to confirm the size and structure of the synthesized AgNPs. The elemental analysis and functional groups responsible for the reduction of AgNPs were analyzed by electron dispersive spectroscopy and fourier transform infra-red spectroscopy Fourier transforms infrared, respectively. A new biological approach was taken by breaking down organic dyes such as methylene blue and congo red. The AgNPs effectively inhibit the fungal growth of *Alternaria alternata*. This could be a significant achievement in the fight against many dynamic pathogens and reduce dye contamination from waste water.

## Introduction

Nanotechnology has emerged in recent years as a promising and versatile technology that deals with the processing of the separation, consolidation and deformation of materials by an atom or by a molecule (Shah et al., 2020). It is a multidisciplinary field that includes the formation, usage, and manipulation of materials with a scale smaller than 100 nm at one dimension (Nakkala et al., 2018), (Raj et al., 2021). Nanoparticles are attracting the attention of current researchers due to their high surface-area-to-volume ratio, which leads to differences in physical and chemical properties compared to bulk counterparts with the same composition (Raj et al., 2021), (Dashora et al., 2022). It opened the doors for rapidly growing technologies because it was involved in the creation and production of innovative materials with unique and superior material properties (Dhand et al., 2016). Metal nanoparticles (such as Ag, Au, and Cu) have been chosen for their exceptional physicochemical properties, including excellent catalytic activity, optical, magnetic, thermal conductivity, biological, and chemical properties, due to the large specific surface area and a high fraction of surface atoms (Rashid et al., 2019), (Seku et al., 2021). Silver is the metal of choice among the various precious metals due to its many unique properties, most notably its powerful antibacterial and catalytic nature. Various physicochemical techniques such as chemical reduction have been used to synthesize AgNPs (Abdelmoteleb et al., 2020). In recent years, conventional approaches to the synthesis of AgNPs have rarely been used. This is due to the use of hazardous compounds in these processes associated with high energy consumption, corrosive nature and expensive further processing (Khanal et al., 2022). Various approaches have been used for the synthesis of metallic NPs, among which the greener route is considered to be an environmentally benign method (Ali et al., 2022). To formulate the NPs, the biological route has received much more attention compared to the rest of the modern techniques (both physical and chemical) due to several important characteristics such as safety, eco-friendly protocols with non-toxic by-products, requirements for gentle reaction conditions and the use of a natural one capping. Biogenic synthesis includes such as fungi (Tyagi et al., 2019), (Osorio-Echavarria et al., 2021), bacteria (Saeed et al., 2020), algae (Bhuyar et al., 2020) and plants (Raj et al., 2018) for the synthesis of AgNPs and found to be a clean, nontoxic and environmentally acceptable technique (Kaur and Jaryal, 2018), (Varadavenkatesan et al., 2020). Plant part extracts such as root, flower and leaves etc (Sahayaraj et al., 2020; Elangovan et al., 2021; Ilahi et al., 2021) are more effective than microorganism-mediated procedures because plants and plant extracts are less sensitive to metal toxicity, cell culture is not required and metabolites such as polyphenols, saponins, tannins, alkaloids, flavonoids, steroids and others are widely available. These metabolites have the ability to stabilize and reduce the silver ions for the synthesis of NPs (Bharathi and Bhuvaneshwari, 2019), (Mosaviniya et al., 2019).

Toxic organic dyes could be used in a variety of industries, including textiles, leather, plastic, paper, paint, cosmetics and pharmaceuticals (Wang et al., 2018), (Singh et al., 2021). Direct contact of the dye with the organisms can lead to serious health problems such as mental health problems, eye damage and central nervous system. The sequestration of synthetic organic compounds from wastewater is a critical environmental issue (Varadavenkatesan et al., 2019). Many physical and chemical methods such as adsorption, ozonation, photocatalysis, microwave-based degradation, chemo-catalysis, Fenton reaction and electrocatalysis can be used to eliminate these pollutants (Nasrollahzadeh et al., 2018). The complete removal of synthetic dyes from wastewater through physical and chemical processes required high technology that is both inefficient and energy-intensive (Bonigala et al., 2018). The removal of hazardous organic pollutants from wastewater using metallic nanoparticles is a unique strategy and a viable alternative to physical and chemical processes (Eisa et al., 2019). AgNPs have been extensively studied among the numerous metallic NPs because of their relatively low cost (Wang et al., 2019). Therefore, a simple and green biosynthetic technique mediated by Plantago ovata leaf extract was developed in this study.

*Plantago ovata* is a member of the Plantaginaceae family and is a winter annual plant known locally as Indian Plantago, Desert Indian Wheat, Isabagol, and Ispaghula. It grows in different regions of the world, mainly in the northern hemisphere between the 26th and 36th latitude (Meyers and Liston, 2008). In India, mainly cultivated in Rajasthan and Gujarat as a crop and for medicinal purposes. Plantago leaves are used for various ailments to treat intestinal disorders and bowel habits, as well as constipation, haemorrhoids, skin irritation, diarrhoea, colon cancer, diabetes, excessive cholesterol and inflammatory bowel diseases such as ulcerative colitis (Ahmadi et al., 2012), (Franco et al., 2020). The plant leaves contain many phytochemicals such as alkaloids, caffeic acid derivatives, coumarins, lipids and oils, mucilage, polysaccharides, sterols and salicylic acid, etc. (Nawaz et al., 2021), (Kumar et al., 2020). These active chemicals could be crucial in reducing silver ions to AgNPs. There is no report on the synthesis of silver nanoparticles using *Plantago ovata* extract and evaluation of their catalytic and antifungal properties, despite the potential of these chemicals in the green synthesis of metal NPs.

Plant pathogenic fungi *Fusarium oxysporum* and *Alternaria alternata* cause various diseases in economically important crops. These phytopathogenic fungi can damage pre- or post-harvest crops as their wide host range can cause a variety of damage depending on the susceptibility of the host, the virulence of the isolate, and the infected regions of the plant (Kgatshe et al., 2019). The use of fungicides to combat fungal diseases is the most effective way. These fungicides are mostly synthetic and have numerous health hazards and multiple environmental and human health side effects (Isa and Lockman, 2019). Various natural and safe substitutes for chemical fungicides have been investigated in recent years. In these, biogenic metal NPs were also used to combat phytopathogenic fungi (Khanal et al., 2022). In the food industry, textile composition, and many environmental applications, AgNPs have been widely used in the form of antimicrobial agents (Mekky et al., 2021). However, few records exist on the potential of AgNPs to combat phytopathogenic fungi.

In this present study, we have reported biogenic and eco-friendly synthesis of silver nanoparticles employing *Plantago ovata* plant extract and characterized by various analytical techniques such as UV-Vis, FT-IR, HR-TEM, FE-SEM, EDS, XRD, AFM and Raman spectra. Further, their catalytic degradation properties against azo dyes MB and CR and *in vitro* antifungal activity against phytopathogenic fungi *Alternaria alternata* and *Fusarium oxysporum* were analysed.

## Materials and methods

### Materials

**FIGURE 1:**
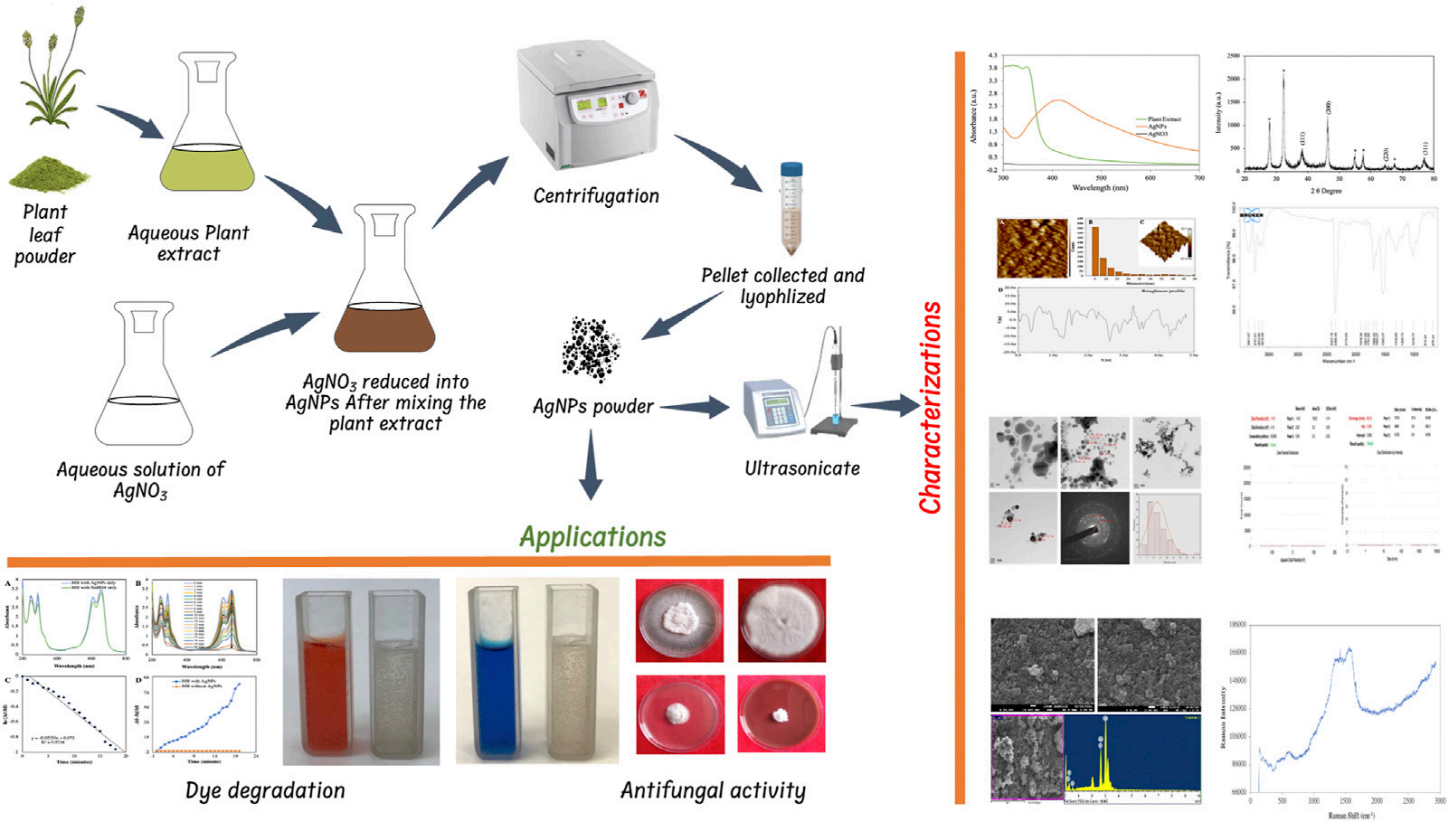
Schematic illustration of the synthesis of AgNPs and their characterization and applications.

All the analytical grade chemicals such as silver nitrate (AgNO_3_), sodium borohydride (NaBH_4_), methylene blue (MB), congo red (CR), potato dextrose agar (PDA), were procured from Sigma-Aldrich, St. Louis, United States and Himedia Pvt. Ltd. New Delhi, India. Fungal culture of *Fusarium oxysporum* (ITCC No. 4998) and *Alternaria alternata* (ITCC No. 6134) were obtained from IARI New Delhi. *Plantago ovata* plants were collected from Sikar district Rajasthan, prepare herbarium and submitted to the herbarium of Rajasthan University Jaipur, Rajasthan, India to obtain voucher number. The voucher specimen (RUBL211771) was retained in the department for future reference. Figure 1 shows the overview of AgNP synthesis, characterization and applications.

### Preparation of the plant extract

Collected plant leaves were washed thoroughly with a tap and followed by double distilled water to remove dust particles and shad dried for 1 week at room temperature. Dried leaves were pulverized with a blender and made into fine powder. Take 5 g of leaf powder mixed with 200 ml sterile deionized water in 500 ml Erlenmeyer flask and boil at 60°C serological water bath for 20 min. After cooling the extract was filtrated through Whatman No. 1 filter paper and stored at 4°C in the refrigerator for further use within a week.

### Synthesis of AgNPs with *Plantago ovata* leaf extract

For AgNPs synthesis 10 ml of plant extract mixed with 90 ml of AgNO_3_ solution was taken from 1 mM stock solution of AgNO_3_ and incubated at 40°C for 24 h. The whole process was done in low light to avoid photo-oxidation of AgNO_3_. The light yellow colour of the solution gradually turn into reddish-brown colour which indicated the reduction of Ag^+^ to Ag^0^. This synthesis of AgNPs was monitored using UV-VIS spectrophotometer scanning between 300 and 700 nm. Afterwards, the solution was centrifuged for 15 min at 10,000 rpm, the supernatant was discarded and pellets were collected. The pellets were washed with de-ionized water and dried in a lyophilizer to make a fine powder.

### Characterization of AgNPs

For the initial characterization of synthesized AgNPs, a UV-Visible spectrophotometer (Systronics 117 UV–VIS spectrophotometer) was used. After 24 h of AgNPs synthesis, absorbance was measured between the wavelength ranges of 300–700 nm with a resolution of 1 nm in a quartz cuvette cell with a 1 cm path length. Further characterization of AgNPs was done using Fourier transforms infrared (FTIR) PerkinElmer Spectrum 2 spectrometer at room temperature in the range 500–4000 cm-1 (PerkinElmer, Inc. Waltham, MA, United States). Crystalline metallic AgNPs were examined using an XRD (Rigaku, Ultima IV, Japan). Powder of AgNPs was analyzed by XRD using CuK radiation (1.54 nm) and a scanning 2θ angle ranging from 20^o^ to 80^o^. The polydispersity index (PDI), zeta size and zeta potential of AgNPs were analyzed using DLS (Malvern Instrument Inc., London, United Kingdom) and were taken at a scattering angle of 90^o^ at a 25°C temperature range. Atomic Force Microscopy (AFM) Multimode Scanning Probe Microscope (Bruker) Germany, was used to analyse surface morphology, and 2D and 3D topography of synthesized AgNPs. The surface morphology of the synthesized AgNPs was carried out using TEM (TEM-Tecnai G2–20, United States) and SEM (JEOL SM-7600F Japan) combined with energy-dispersive X-ray spectroscopy (Oxford EDS system) from Sophisticated Analytical Instrument Facility (SAIF), IIT Bombay. Raman Spectroscopy was used to detect the AgNPs molecular structure and chemical bonds were carried out using lso Plane SCT-320, PIXIS 100 Princeton Instrument from MNIT Materials Research Centre (MRC), Jaipur, Rajasthan, India.

### Catalytic degradation of dyes

The degradation of organic dyes MB and CR was carried out by using biosynthesized AgNPs as a catalyst. Before commencement, the AgNPs were dispersed in deionized water using an Ultra probe sonicator for 10 min and a stock solution for dye was prepared. The 3 ml reaction mixture contains desired volume of dyes, NaBH_4_ and AgNPs as mentioned in Table 1. The progress of the reaction was monitored by using the UV–vis spectrophotometer (Systronics 117 UV–VIS spectrophotometer). The absorbance of each dye was measured at 1 min time intervals and the change in absorbance at each time interval was examined for kinetic study under different conditions. Reaction mixture with AgNPs and NaBH_4_ separately used as control. A reaction mixture without AgNPs was used as a reference and pseudo-first-order kinetics was analyzed to evaluate the rate constant as per the following equation:

$$In\left(A_{t}/A_{0}\right)=-k_{t}$$ (1)

**TABLE 1:** Detail of experiment for catalytic degradation of dyes.

Dye^’^s	Dye’s conc. (M)	Volume of dye’s (µL)	Volume of H_2_O (ml)	NaBH_4_ (0.05M)	Volume (ml) of AgNPs(0.05%)	R (µL)eduction time (min)	Rate constant (K)
M (min)B	0.01	90	2.5	0.4	10	20	0.056^–1^
CR	0.01	80	2.5	0.4	20	18	0.166^–1^

The % degradation of the dyes was estimated through the following equation

$$Percent\;degradation=A_{0}-A_{t}/A_{0}\times 100$$ (2)

Where, A_0_ is the initial absorbance of dye, A_t_ is the absorbance of dye at time t and k is the rate constant. The whole reaction of degradation was processed at room temperature.

### Antifungal activity of synthesized nanoparticles

The antifungal activity of AgNPs against *A. alternata* and *F. oxysporum* was carried out using the poison food method (Grover and Moore, 1962). AgNPs at different concentrations (25, 50, 75 and 100 μg/ml) were added to 20 mL of sterilized and cooled autoclaved PDA medium along with synthetic fungicides Bavistin and Mancozeb at 100 μg/ml, plant extract and water as control. After the media solidify the mycelial disc of 6 mm diameter from 7 days old pure culture was placed at the centre of each Petri plate and incubated at 28 ± 2°C. After 7 days of incubation, observations were recorded of fungal mycelium growth inhibition and the percentage of inhibition of fungal growth was calculated as per the following formula given by Vincent (Vincent, 1947).

$$\%Mycelium\;inhibition=\frac{Gc-Gt}{Gc}\times 100$$ (3)

Where *Gc* is the diameter of test fungal mycelia growth in a control plate and *Gt* is the diameter of test fungi mycelia growth in a treated plate.

### Spore germination effect

The activity of spore germination inhibition of AgNPs has been assayed against *F. oxysporum* and *A. alternata* fungi as described by Saharan et al., 2015 (Saharan et al., 2015). Different concentrations of AgNPs (25, 50, 75 and 100 μg/ml), 100 μg/ml of AgNO_3_, Bavistin and Mancozeb, and water as control were used to determine spore germination activity. The spore suspensions were made in sterilized distilled water containing 1×10^3^ spores/ml. On the glass depression slide (Himedia, Mumbai, India), 50 μl of test fungi suspensions and 50 μl of different concentrations of AgNPs were also added in the same cavity. To maintain the required humidity, each cavity slide was incubated at 28 ± 2°C for 12 h. Three replicates of each treatment, including standard, were maintained and observed under a microscope to calculate spores germination inhibition rate.

$$\%spore\;inhibition=\frac{Number\;of\;spore\;germinated}{Total\;number\;of\;spore\;examined}\times 100$$ (4)

### Statistical analysis

Statistical analysis was performed using analysis of variance (ANOVA), followed by a Tukey HSD test (*p* = 0.05) using SPSS Version 26. The Microsoft Office, OriginPro 2020 and Corel Draw software were used for calculation and designing Graphs and figures.

## Results and discussion

### UV–visible spectroscopy

**FIGURE 2:**
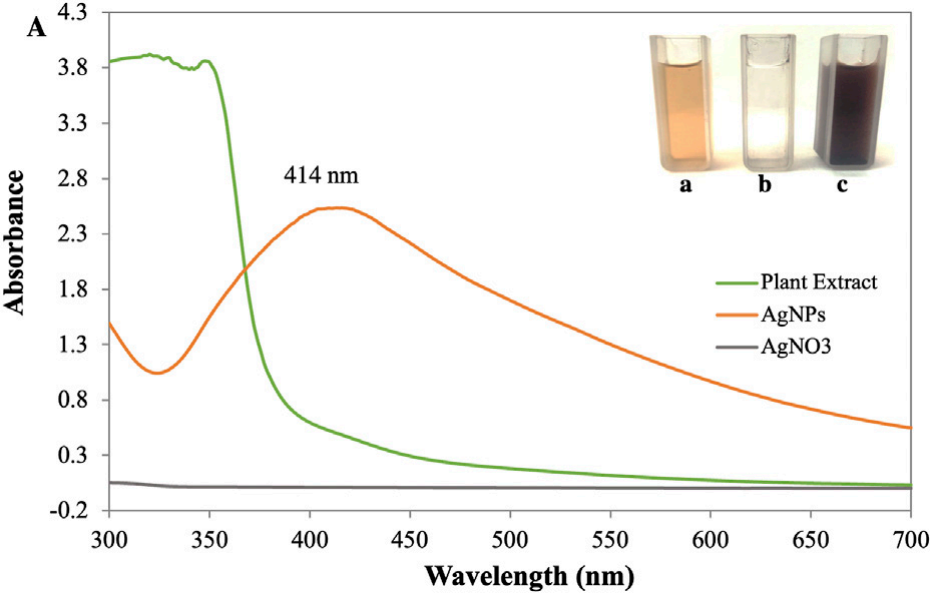
**(A)** UV-visible spectra of synthesized AgNPs [Inlet (a) AgNO3 (b) Plant Extract (c) AgNPs].

The primary investigation of AgNP synthesis was confirmed by UV-Vis absorption spectra scanning between 300 and 700 nm. Deionized water was used as a reference. The peak of the synthesized AgNPs was observed at 414 nm (Figure 2). It was shown that the phytocompounds contained in the leaf extract successfully reduced the Ag + ions to AgNPs since it is known that colloidal silver shows a typical absorption band in the range of 400–450 nm due to the surface plasmon resonance of the conducting electrons of the metal (David and Moldovan, 2020). During the process of AgNPs synthesis, adding a reducing agent, *P. ovata* aqueous extract in AgNO_3_ solution resulted in a colour change from pale yellow to reddish-brown was observed. However, the reduction of silver salt was initiated as the increasing pH of the aqueous extract towards slight alkaline (pH 9). These indicated that the pH plays a crucial role in reducing silver salts to silver ions (Parthiban et al., 2019). It has been revealed by many previous studies that alkaline pH can efficiently reduce the nanoparticles and further encapping them. In their research on the impact of pH on the synthesis of nanoparticles, Sathishkumar et al. (2009) noted the formation of small, stable nanoparticles at an alkaline pH (Sathishkumar et al., 2009). They claim that lower pH values encourage the nucleation of nanoparticles whereas higher pH results in electrostatic repulsion, which causes the creation of smaller nanoparticles. The functional groups in the plant extract have reduced power, which is less active at acidic pH, but it becomes more active as the pH rises to basic pH, resulting in an increase in their reductional power and the development of thermodynamically advantageous structures. Alkaline conditions are therefore favourable for biosynthesis.

### FTIR spectroscopy

**FIGURE 3:**
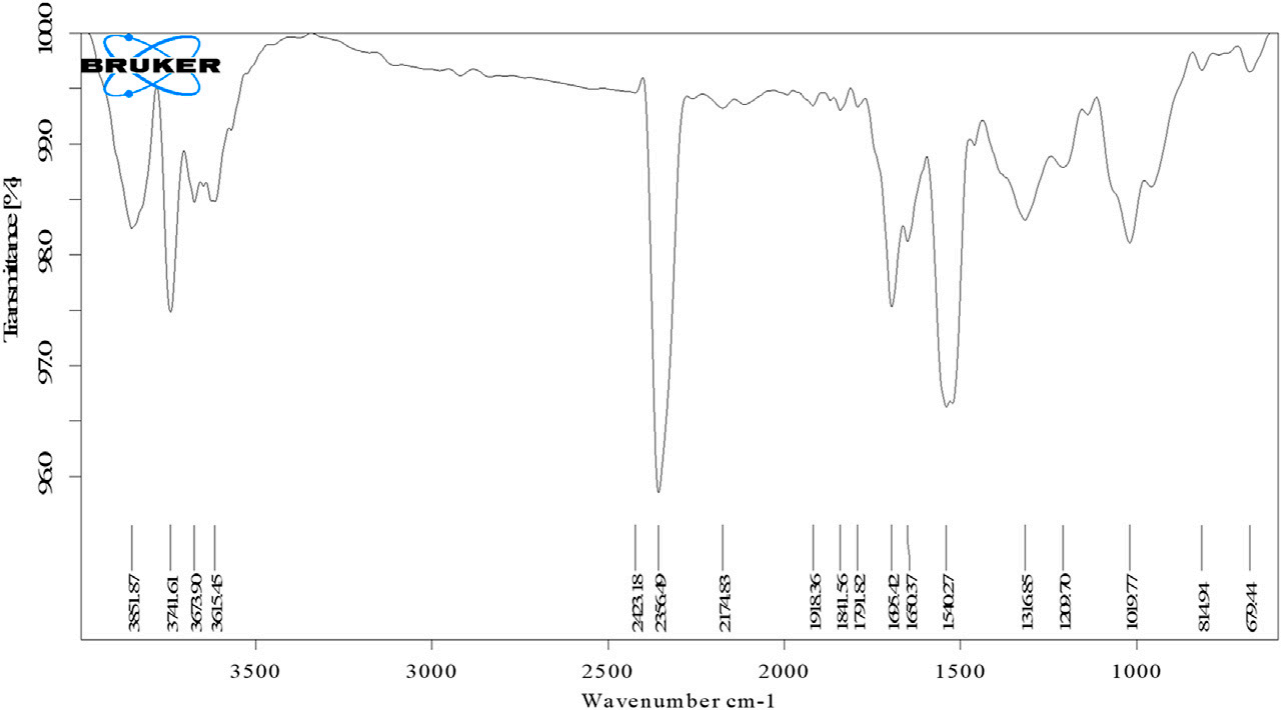
FTIR spectra of synthesized AgNPs.

FTIR spectroscopy was performed to identify the functional groups associated with the biomolecule involved in AgNP reduction and stability shown in Figure 3. The significant absorption bands at 3,702.37 and 3,332.82 cm^−1^ correspond to OH and C-H stretches, indicating that alcohol and alkyne are present (Hasnain et al., 2019). Similarly, prominent peaks appeared in the IR spectra at 1,594.97 and 1,384.51 cm^−1^ ascribed to C=C stretches of cyclic alkenes (aromatic compounds) and C-H stretches of the amide II bond, which confirmed the presence of proteins (Al-Nuairi et al., 2020). Peaks at 1,075.68 cm^−1^ relate to C-O stretching of the carboxyl ether group and peaks at 984.20 cm^−1^ are due to C-OH stretching of the carboxyl ether group (Nouri et al., 2020). The C-H stretch of the aromatic group was assigned to the peak at 856.97 cm^−1^ while the OH bend of the phenol group was assigned to the peak at 540.81 cm^−1^ (Hawar et al., 2022). These distinct absorption peaks indicated the presence of phytochemicals from extracts (such as amino acids, carbohydrates, phenols, flavonoids, alkaloids, terpenoids, proteins, and water-soluble biomolecules, among others) on the surface of NPs, which were responsible for capping and efficient stabilization of AgNPs and prevention of aggregation of AgNPs (Jemilugba et al., 2019). The morphology (size, shape) and overall functional properties of biosynthesized AgNPs are significantly influenced by the nature of the biomolecules involved in silver ion capping and reduction (Sharma et al., 2020a). According to FTIR analysis, the aromatic compounds (C=C), ether groups (C-O), hydroxyl (-OH) and carboxyl groups (C-OH) in the leaf extract are most likely involved in the reduction.

### X-ray diffraction

**FIGURE 4:**
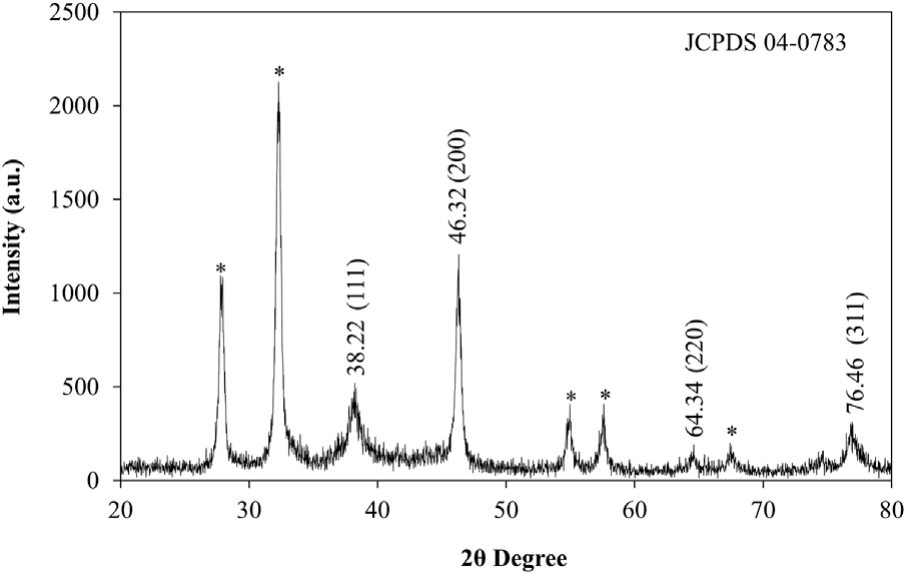
XRD pattern of synthesized AgNPs.

XRD patterns were used to analyze the crystalline nature, structure and size of synthesized AgNPs from *Plantago* leaf extract. The XRD pattern showed the Bragg reflection plane in the 2θ range of 20–80° and prominent peaks at two values, which can be attributed to the 111, 200, 220, and 311 crystal planes of AgNPs, at 38.22, 46.32, 64.34 and 76.46 shown in Figure 4. These peaks signify that the silver particles with a crystallite size of 17.09 nm have a face-cantered cubic structure and the results are comparable with TEM data (Gulbagca et al., 2019). Other peaks were observed for the 2θ values, which are due to residues of different organic components of the plant extract. These peaks indicated that various plant metabolite units are crystallized on the surface of AgNPs, consistent with the results of Saba Pirtarighat et al. (Pirtarighat et al., 2019). The Braggs diffraction peaks were observed by XRD analysis, which agreed well with a database of the Joint Committee on Powder Diffraction Standard of Ag (JCPDS Card No. 04-0783). There were few unassigned (*) peaks, possibly due to the crystallization of the bioorganic moieties on the surface of the AgNPs, as noted in many previous studies (Raj et al., 2020). From the X-ray magnification, the size of the crystalline NPs was evaluated by the Scherrer equation.

D=kλ/βcos⁡θ (5)

Where D is the average crystal size, k is a constant equal to 1, the source of the X-rays is (λ = 1.54), the angular line is full width at half maximum (FWHM) and is the Bragg angle. AgNPs were found to have an average crystal size of 17.09 nm.

### DLS analysis

**FIGURE 5:**
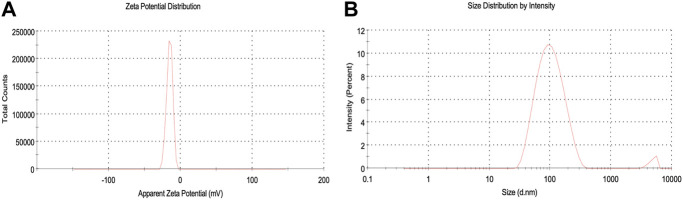
DLS analysis **(A)** Zeta potential, **(B)** Zeta size of synthesized AgNPs.

The hydrodynamic size (size and surface water molecule), surface potential, and colloidal stability of biosynthesized AgNPs were studied using the dynamic light scattering (DLS) technique. Before analysis, samples were highly dispersed in water using ultrasound to keep the polydispersity index (PDI) below 0.5, which is much needed during analysis (Mekky et al., 2021). The observed negative potential of biosynthesized AgNPs was 14.9 mV. The negative value could be due to the capping effect of biomolecules present in the leaf extract of *P. ovata*, which supports the high stability of AgNPs (Figure 5A) (Bharathi and Bhuvaneshwari, 2019). The stability of the synthesized AgNPs was due to the same negative charge on the particles creating a repulsive force between the particles, keeping them suspended in the aqueous solution (Raj et al., 2018). The average size of the AgNPs was 88.3 nm (see supplementary). The polydispersity index (PDI) was estimated to be 0.284, indicating the narrow distribution of the AgNPs (see supplementary). The particle size in DLS appeared larger than in TEM because DLS is measured based on the hydrodynamic diameter (size and surface water molecule) of the particles and gives an intensity-weighted average particle size, while the size obtained from TEM is based on the dry particle diameter and gives an average particle size. (Nakkala et al., 2018). In addition, agglomeration or poor dispersion significantly increases the mean value and thus generally contradicts the SEM/TEM results (Shah et al., 2020).

### AFM analysis

**FIGURE 6:**
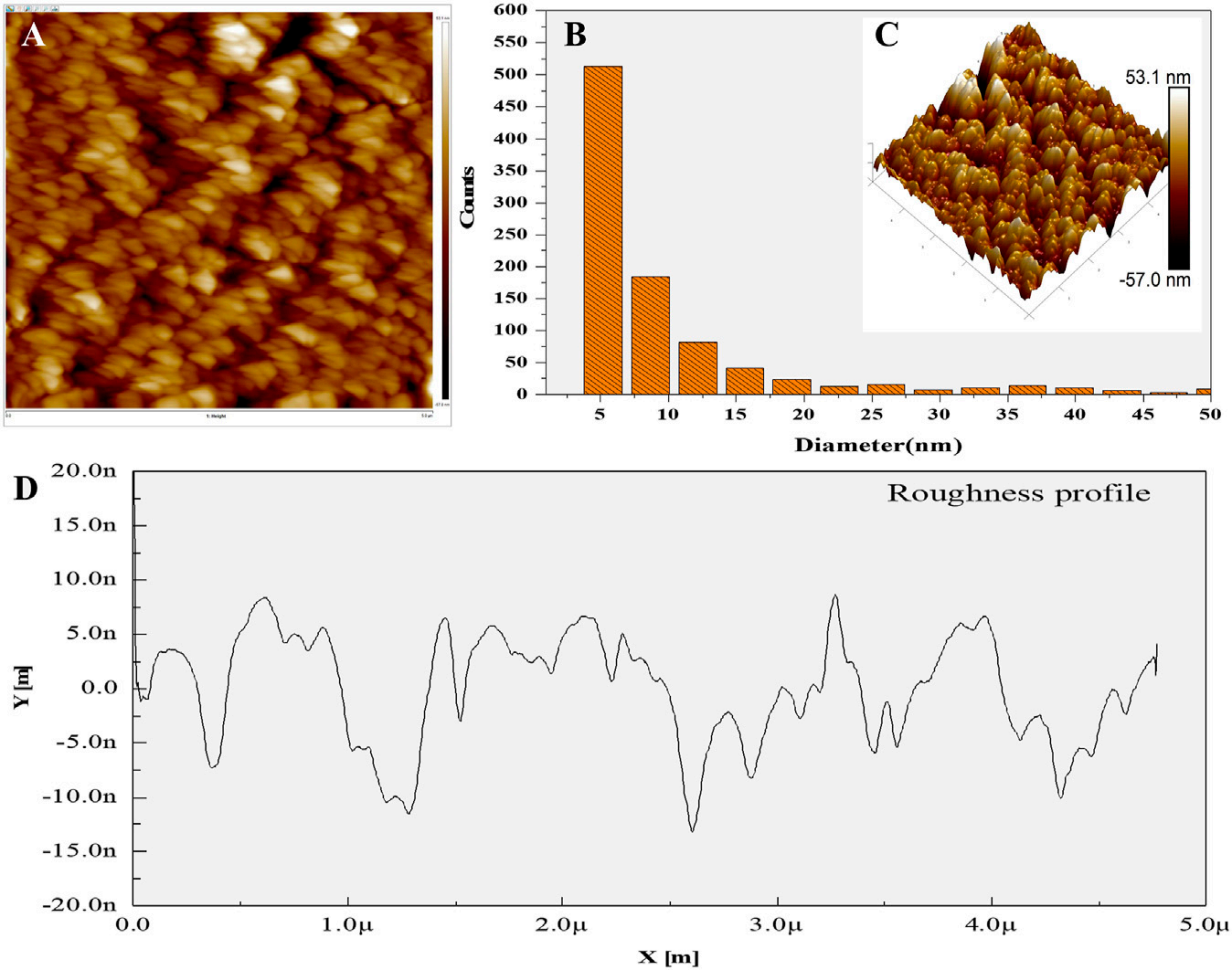
AFM analysis **(A)** 2D image **(B)** Histogram, **(C)** 3D image, **(D)** Surface roughness.

AFM data delineated the biosynthesized AgNPs were roughly spherical with an average grain size of 14.56 nm and root mean square roughness is 10.97 nm. The figure shows the two-dimensional (Figure 6A), three-dimensional (Figure 6C), histogram of sizes obtained from AFM analysis Figures 6B, D shows the roughness profile from the 3D image of synthesized AgNPs calculated. The maximum height of the profile was 37.76 nm, the maximum roughness of valley depth was 13.13 nm and the average maximum height of the roughness was 24.63 nm. These results were consistent with previous studies on the surface morphology of AgNPs in AFM analysis (Bhat et al., 2021), (Yugandhar and Savithramma, 2016). It supports the existence of nanostructures, their fairly homogeneous distribution and the absence of significant agglomeration.

### TEM and SEM-EDX analysis

**FIGURE 7:**
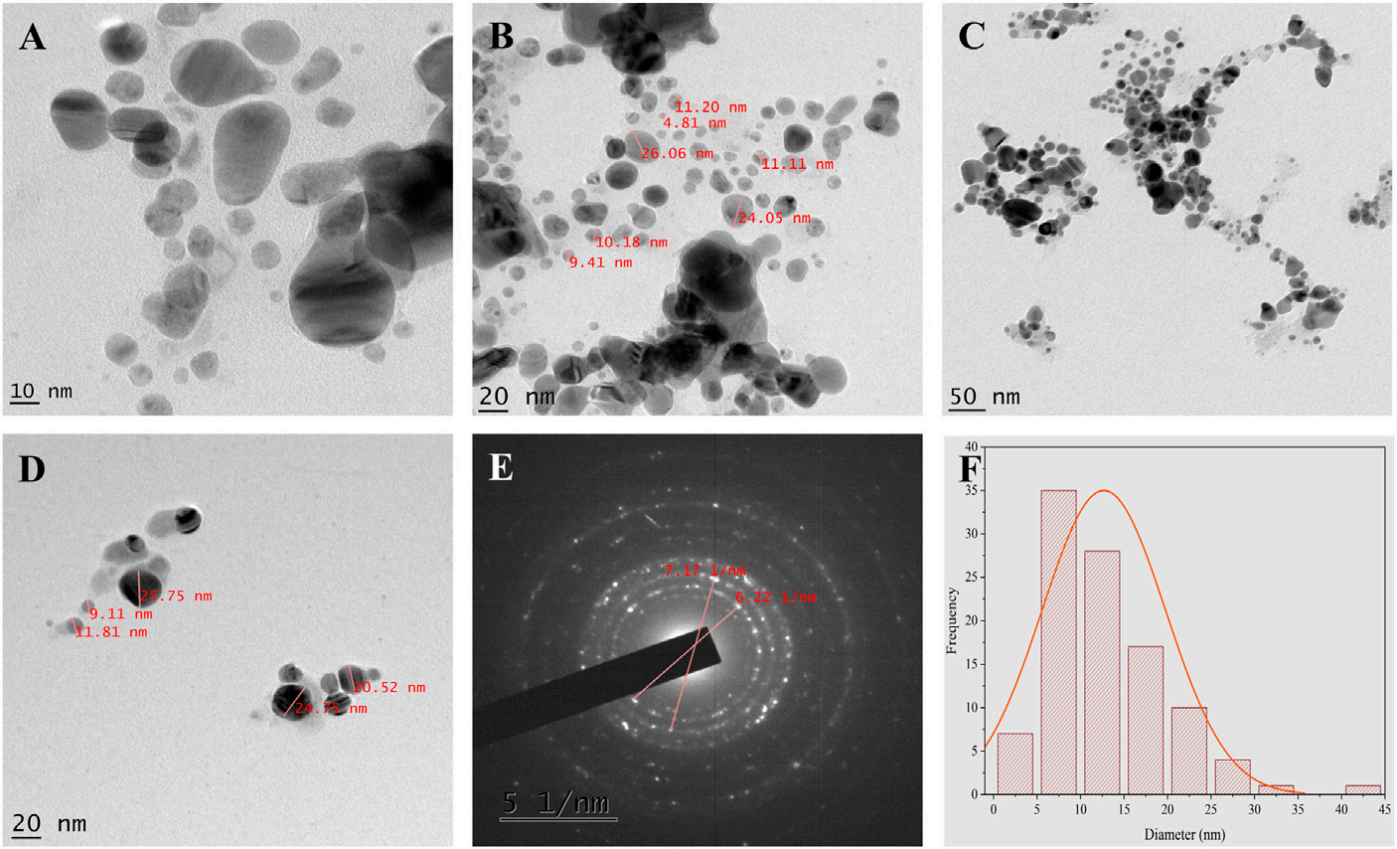
TEM analysis **(A–D)** TEM micrographs of AgNPs at different magnification **(E)** SAED pattern **(F)** Histogram shows average particle size distribution of Synthesized AgNPs.

**FIGURE 8:**
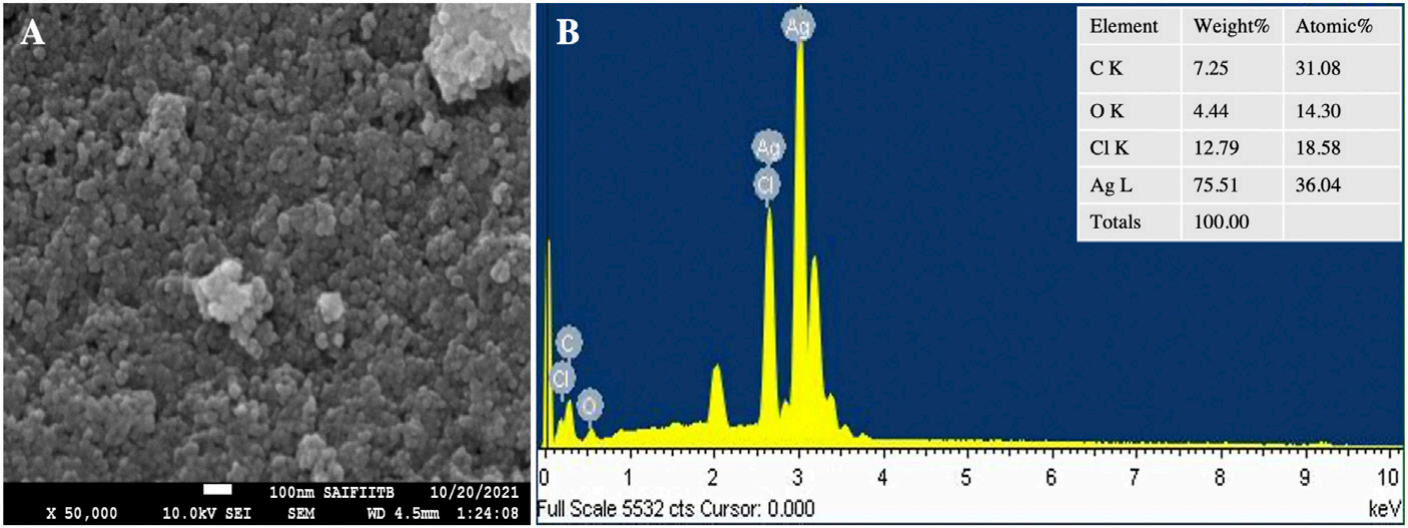
SEM analysis **(A)** SEM micrograph **(B)** EDS spectrum of synthesized AgNPs.

TEM images (Figure 7) show fine silver NPs that are roughly spherical, have a smooth surface and are well scattered with a tight, compact arrangement in TEM images, consistent with SEM results. From the TEM figures, it was observed that the particles were predominantly spherical, discrete, polydisperse, and distorted spherical shapes with uniform distribution (AryanRubyMehata, 2021). The calculated value of the biosynthesized AgNPs agreed with the selected area electron diffraction (SAED) pattern and resembled a good agreement with the XRD results (Figure 7E). The SAED image shows distinct and brilliant points in a circular ring, arising from the numerous crystallites of the diffraction planes of the face-cantered cubic crystals (111, 200, 220, 311) (Khan et al., 2020). According to FE-SEM analysis, the particles are consistently spherical and have an organic layer coating their rough surface that serves as a capping agent (Figure 8A) (Bhat et al., 2021). The TEM and FE-SEM analyses were used to determine the particles’ sizes, which are primarily in the range of 10–30 nm with an average size was 12.67 nm. The particle size distribution graph is based on fitting the histogram to a Gaussian model as shown in Figure 7F. In addition, the particle size calculated from the histogram agrees closely with the TEM images.

The elemental composition of the reaction mixture was confirmed by EDX. The horizontal axis shows the energy in keV and the vertical axis shows the number of X-ray counts. The EDX result shows (Figure 8B) the presence of silver (75.51%) with a significant peak as well as the additional elements C (7.25%), O (4.25%) and Cl (12, 79%) in weight per cent and Ag (36.04%), C (31.04%), O (14.30%), and Cl (18.58%) in atomic per cent. No ionic silver peak is observed in the EDX spectrum, confirming the bio-reduction of Ag^+^ to stable Ag^0^ in satisfactory yield. Therefore, according to the observed EDX patterns, the biosynthesized AgNPs are highly pure and crystalline due to the complete bio-reduction of silver ions caused by phytochemicals present in the aqueous extract of *P. ovata* leaves. The C, O and Cl signals are mainly attributed to the biomolecules in the *P. ovata* leaf extract (Hambardzumyan et al., 2020), (Vijayan et al., 2018). The presence of elemental silver as the main ingredient was verified by EDX analysis, which was possible due to the successful conjugation of *P. ovata* to AgNPs.

### Raman spectra analysis

**FIGURE 9:**
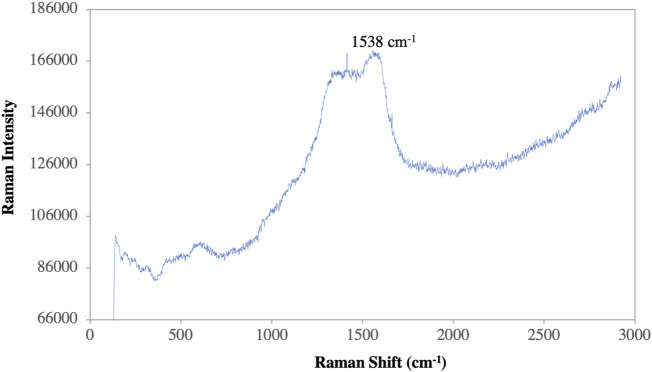
Raman spectroscopy of synthesized AgNPs.

A Raman spectroscopy study was performed to investigate the detailed interactions between capping components and AgNPs and to examine the functional groups (Bélteky et al., 2019). The significant absorption bands at 2,847 cm^−1^ correspond to the C-H stretching vibrations and show the hydroxypropyl group shown in Figure 9. Symmetric and asymmetric vibrations of C=O bonds were observed at the peaks 1,330 cm^−1^ and 1,538 cm^−1^. This refers to the presence of essential amino acids with aromatic side chains (Abdel-Azeem et al., 2020). The bands at 1,538 cm^−1^ and 1,330 cm^−1^ indicate the presence of AgNPs. The other peak obtained at 562 cm^−1^ could be related to the bending vibrations of N-H associated with the amide structure (Katta and Dubey, 2021). The interaction between the leaf extract and AgNO_3_ may be seen in the Raman spectra of produced AgNPs, which have a peak at 335 cm^−1^. The existence of the silver lattice vibrational models is indicated by the band at 152 cm^−1^ (Kgatshe et al., 2019).

### Catalytic activity

**FIGURE 10:**
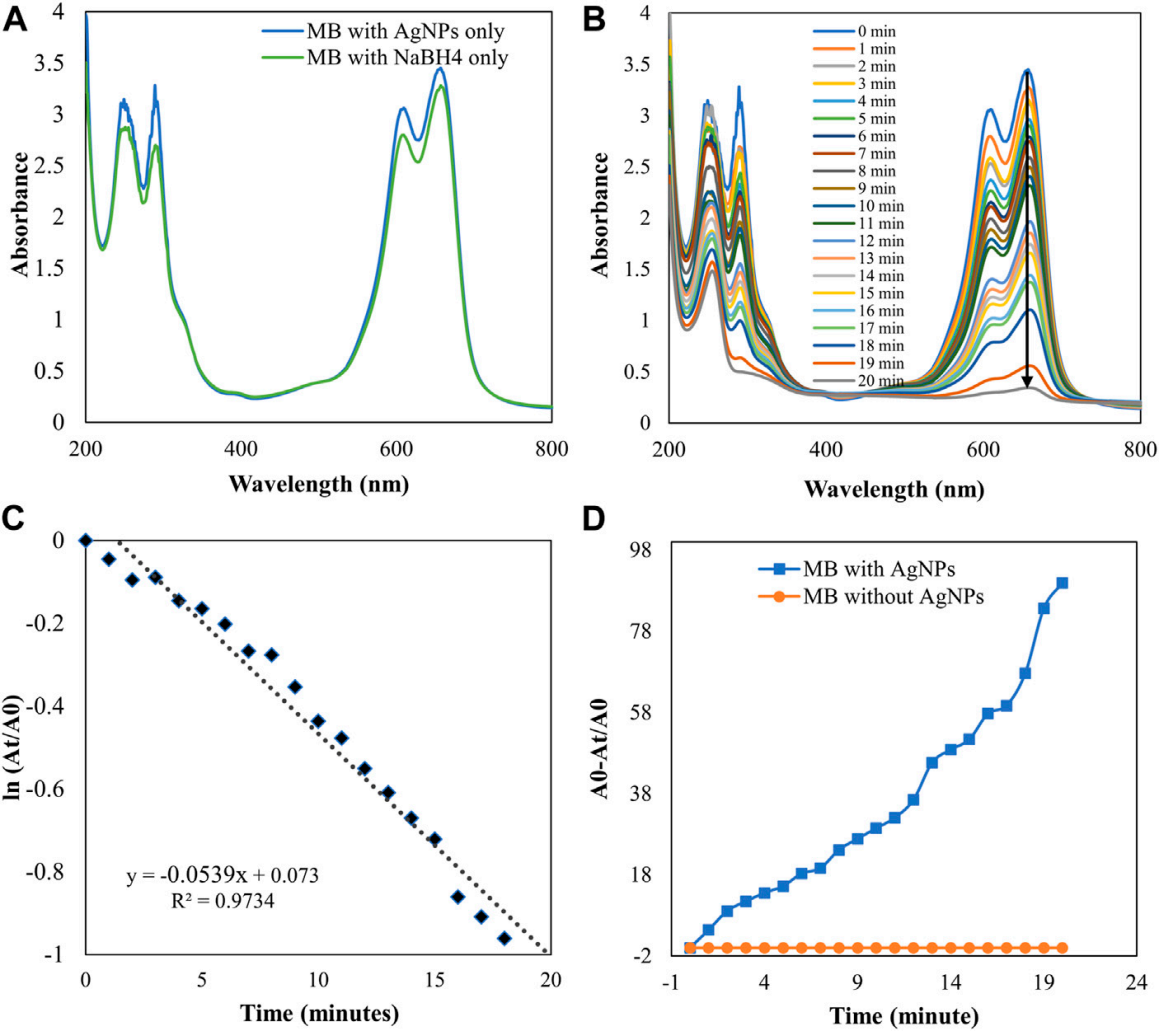
UV-visible absorption spectra **(A)** MB with AgNPs and NaBH_4_ separately **(B)** Catalytic degradation of MB by NaBH_4_ in the presence of AgNPs, **(C)** Pseudo-first order plot of ln (A_t_/A_0_) vs time of MB, **(D)** Percent degradation of MB with time by AgNPs.

**FIGURE 11:**
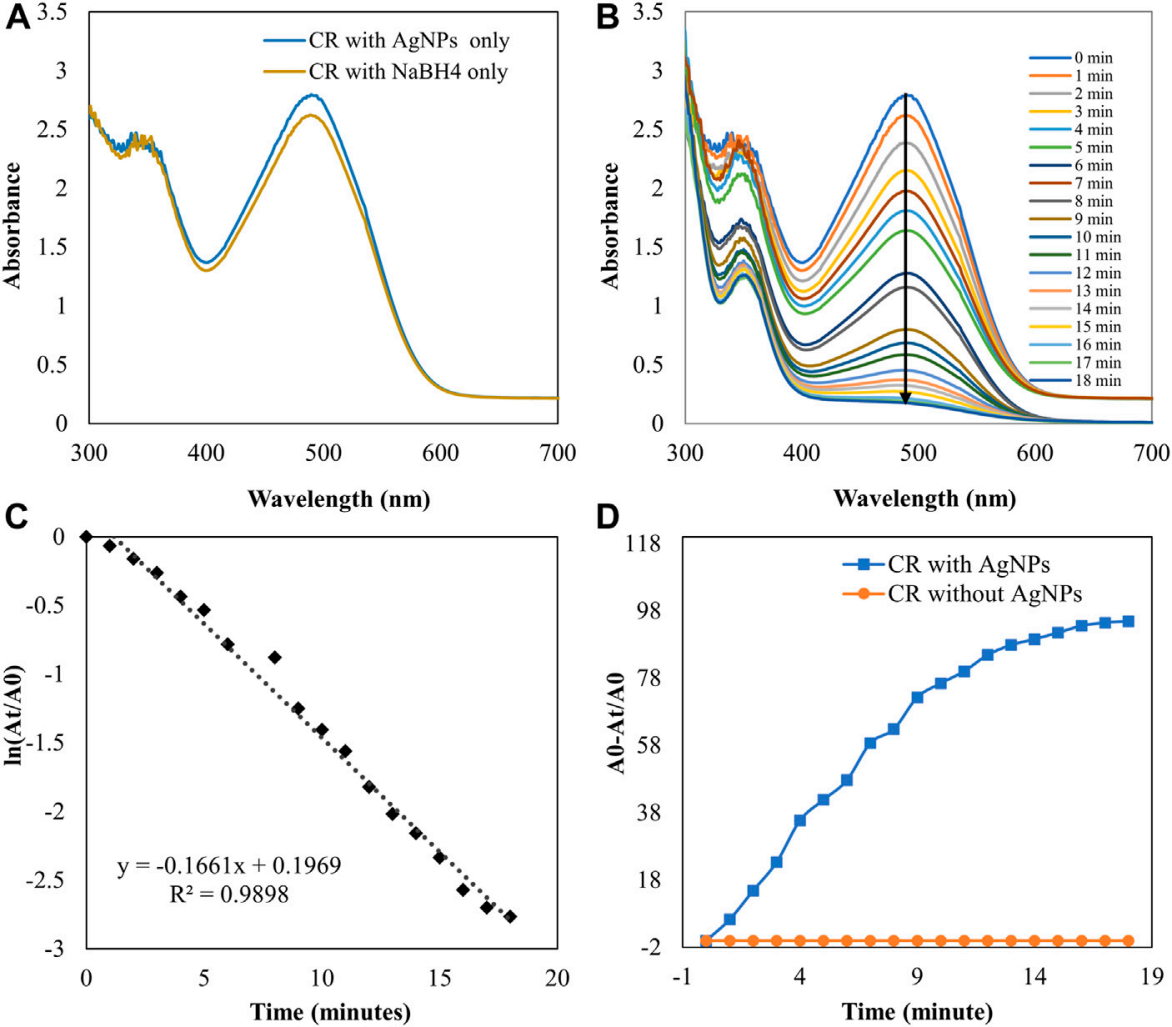
UV-visible absorption spectra pf **(A)** CR with AgNPs and NaBH_4_ separately **(B)** Catalytic degradation of CR by NaBH_4_ in the presence of AgNPs **(C)** Pseudo-first order plot of the ln (A_t_/A_0_) vs. time of CR **(D)** Percent degradation of CR with time by AgNPs.

The degradation of MB was performed by AgNPs synthesized from P. ovata leaf extract. The highest absorption value of pure MB is 664 nm, which corresponds to the MB n→π* transition (Figure 10A). AgNPs and their composites have a larger surface area than other NPs, resulting in higher catalytic activity in dye removal and degradation processes. Norain et al. investigated the reduction of MB by biosynthesized AgNPs (Isa and Lockman, 2019). During the process, slow reduction was seen in the presence of the powerful reducing agent NaBH_4_. After the addition of an amount of biosynthesized AgNPs in the dye solution in the presence of NaBH_4_, the dye gradually decreases and then shifts towards a higher wavelength. The reduction in absorbance indicates that biosynthesized AgNPs are capable of degrading MB. The reaction is completed in 20 min and MB colour is completely degraded. Which shows a significant decrease in MB absorbance and an increase in the SPR peak of AgNPs at 664 nm shown in Figure 10, Which frequently shows the electron relay effect in which electron transfer occurs between leaf extract mediated AgNPs and MB dye. The linear expression of Lagergren pseudo-first order for MB adsorption by *P. ovata* mediated AgNPs is shown in Figure 10C. The absorption of biosynthesized AgNPs with a rate constant of 0.056 min^−1^ is interpreted by our results. Results matched with *Catharanthus roseus* leaves mediated AgNPs and their efficiency in catalytic and adsorption kinetics investigations of MB dye reported in a previous study (Anjum and Riazunnisa, 2021). For the breakdown of colours, most industrial chemists use NaBH_4_, a powerful reducing agent. In the presence of NaBH_4_ and AgNPs separately, the reaction proceeded slowly and only a small change in absorbance was observed, as shown in Figures 10A, 11A (Albeladi et al., 2020).

The catalytic degradation of carcinogenic CR dye using *P*. *ovata* leaf extract mediated AgNPs in the presence of NaBH_4_ was investigated in this work. Because of their complex structure and the inclusion of the diazo group, which provides them with physicochemical, thermal and optical stability and because of health concerns, CR is difficult to biodegrade. Aqueous CR electronic spectra revealed two absorption bands at 498 (π→π*) nm and 348 (π→π*) nm (Figure 11) (Varadavenkatesan et al., 2020). During the process after a long period, NaBH_4_ alone was unable to adequately reduce the CR dye. The addition of *P*. *ovata* mediated AgNPs to the reaction mixture, on the other hand, resulted in CR dye degradation in just 18 min. The azo bonds (-N=N-) in the dye molecule are destroyed during the CR reduction process, resulting in a variety of aromatic amine derivatives. Abolanle et al., 2020 noted a constant decline in the absorption band strength at 498 and 348 nm as time progressed and the colour changed from radish brown to colourless (Tito et al., 2020). The link between ln (A_t_/A_0_) and response time was discovered to be linear. The rate constant k was determined to be 0.166 min^−1^ at room temperature and it follows pseudo-first-order kinetics. The biologically synthesized AgNPs have a lot of promise as catalytic agents for purifying organic dye-contaminated wastewater and industrial effluents because of their stability and strong catalytic activity (Seku et al., 2021). Similar findings from other previous studies on the dye degradation activity of synthetic AgNPs are presented in Table 2.

**TABLE 2:** Previous studies of Dye degradation activity of synthesized AgNPs.

Plants	Catalyst	Reaction time (Min.)		References
		CR	MB	
*Eulophia herbacea*	AgNPs	30	30	Pawar and Patil, (2020)
*Citrus paradisi, Mentha aquatica*	AgNPs	9	26	(Naseem et al., 2020), (Nouri et al., 2020)
*Manilkara zapota*	AgNPs	40	30	Sharma et al. (2020b)
*Bryonia alba*	AgNPs	40	36	Nasrollahzadeh et al. (2018)
*Punica granatum, Laurus nobilis*	AgNPs	8	60	(Banu et al., 2020), (Kucukcobanoglu et al., 2021)
*Prosopis juliflora, Terminalia bellerica*	AgNPs	20	60	(Arya et al., 2018), (Sherin et al., 2020)
*Plantago ovata*	AgNPs	18	20	This work

**FIGURE 12:**
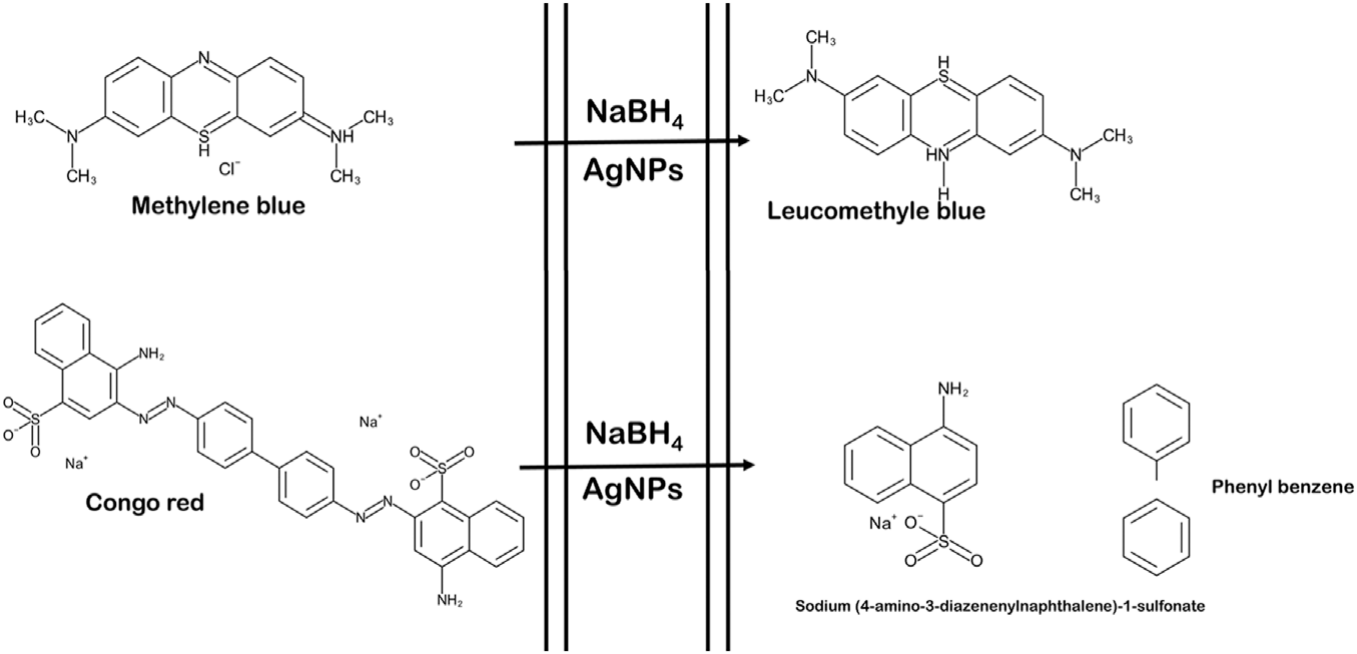
Schematic representation of reaction of dye degradation (the figure is adapted from our previous studies Raj et al., 2020).

As a result, the Langmuir–Hinshelwood model suggested that the catalytic degradation of organic dyes was due to a surface reaction between reactant and AgNPs (Figure 12) (Raj et al., 2020). NaBH_4_ serves as both an electron donor and a hydrogen supplier in this scenario. AgNPs act as an intermediate to transfer the electrons between BH_4_^−^ ion and dye because of their high negative potential (Edison and Sethuraman, 2012), (Edison et al., 2016). After adding NaBH_4_ to a solution having dye and AgNPs, the BH_4_^−^ ion from NaBH_4_ and dye molecules adsorb on the AgNPs surface, resulting in instantaneous electron and hydrogen transport. Diffusion among adsorbed molecules causes desorption of the colourless degraded by-product that might give additional catalytic sites for the breakdown of MB attributable to AgNPs’ wide surface area.

### Antifungal assay of silver nanoparticles

**FIGURE 13:**
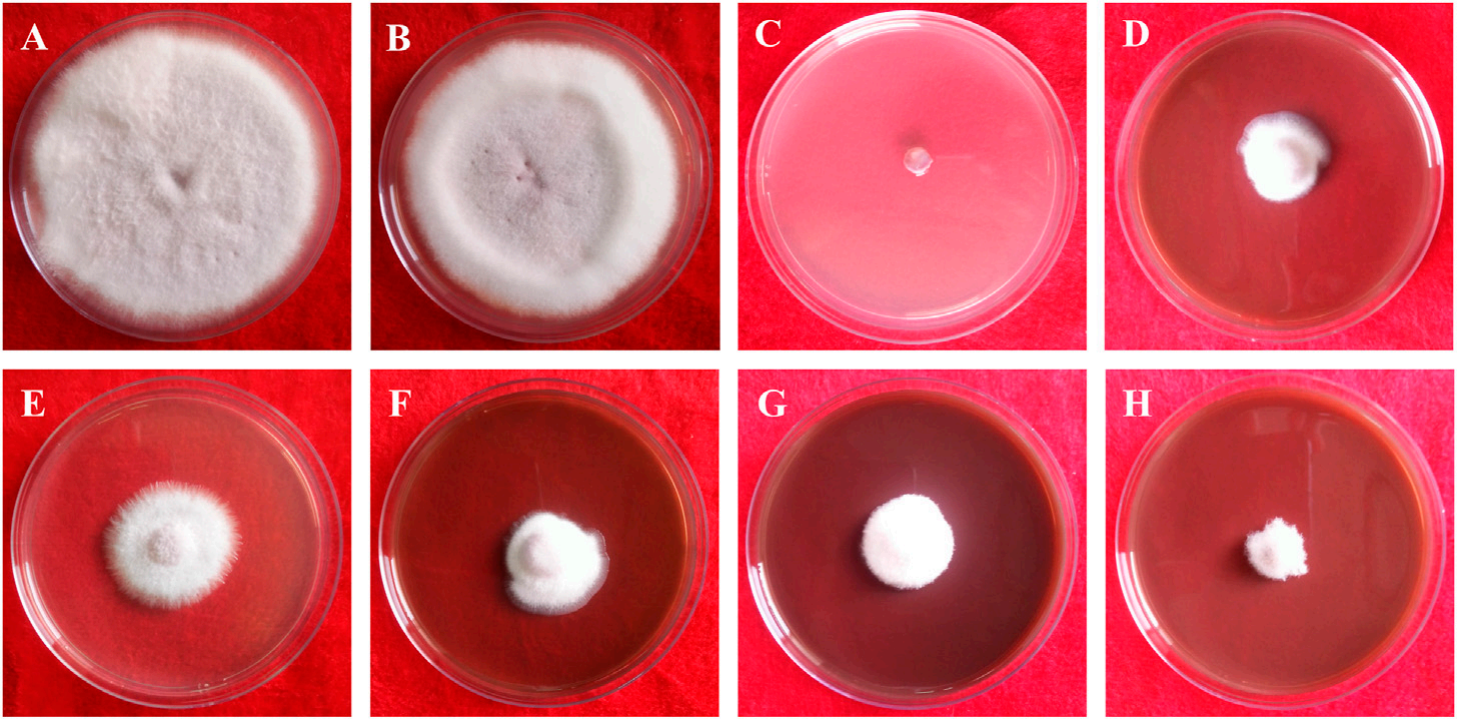
*In vitro* antifungal of synthesized AgNPs against *Fusarium oxysporum*. **(A)** Control, **(B)** Plant extract **(C)** Bavistin, **(D)** AgNO_3_, **(E–H)** 25, 50, 75, 100 μg/ml AgNPs.

**FIGURE 14:**
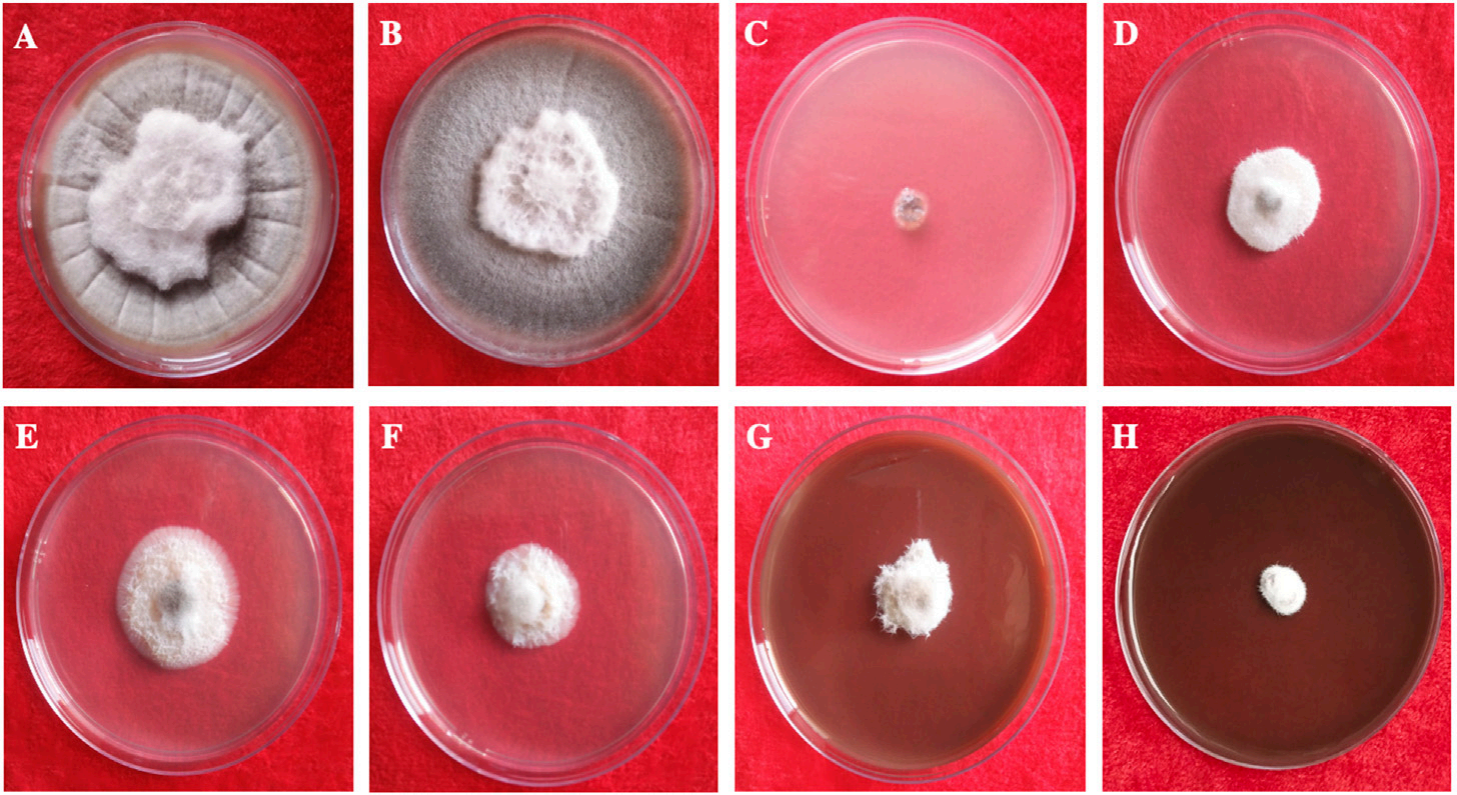
*In vitro* antifungal of synthesized AgNPs against *Alternaria alternata*. **(A)** Control, **(B)** Plant extract **(C)** Bavistin, **(D)** AgNO_3_, **(E–H)** 25, 50, 75, 100 μg/ml AgNPs.

This study clearly showed that AgNPs have good antifungal activity against *F. oxysporum* and *A. alternata* (Figures 13, 14). However, the reduction in mycelial growth was observed slightly more in *A. alternata* than in *F. oxysporum*. *Fusarium*, *Alternaria*, AgNPs concentration and their interaction were very significant source of variation in fungal growth on PDA. The concentration of AgNPs was the most important source of variation, according to the relative contribution, whereas for the control treatment of both fungal strains, the interaction was the least important subject. Regardless of the culture medium, all AgNPs concentrations (25, 50, 75, and 100 μg/ml) were capable of suppressing fungal growth shown in Table 3. As the concentration of AgNPs increases, inhibition generally increased. At a concentration of 100 μg/ml, fungal growth was drastically retarded (Dawoud et al., 2021). As a positive control, the commercial fungicide Bavistin (100 μg/ml) showed 100% inhibition of fungal mycelial development as it is a good antifungal agent but have toxic effects on human. Whereas AgNO_3_ (100 μg/ml) showed 63.67 ± 2.30 inhibition for *F*. *oxysporum* and 60 ± 1.11% for *A*. *alternata* and plant extract was found ineffective in inhibiting mycelia growth of both fungal strains. This dimension allows these NPs to readily enter, aggregate, and interact with cell membranes, thereby inactivating the protein activities that contribute to cell death (Nguyen et al., 2020), (Al-Otibi et al., 2021a).

**TABLE 3:** Mycelial growth inhibition activity of synthesized AgNPs.

Treatment (µg/ml)	% Inhibition (mycelial growth) *Fusarium* oxysporum	% Inhibition (mycelial growth) *Alternaria alternata*
Control	0.00 ± 0.00f	0.00 ± 0.00g
Plant extract	1.48 ± 1.70f	0.74 ± 0.64g
Bavistin	100.00 ± 0.00a	100.00 ± 0.00a
AgNO_3_	59.63 ± 2.57d	60.00 ± 1.11e
AgNPs		
25 μg/ml	46.67 ± 4.01e	52.96 ± 1.70f
50 μg/ml	57.41 ± 2.80d	71.48 ± 1.28d
75 μg/ml	70.74 ± 1.70c	79.26 ± 1.31c
100 μg/ml	81.48 ± 0.64b	86.67 ± 1.11b

Values are means of three independent replicates (n = 3).±Indicate standard errors. Means followed by the same letter(s) within the same column are not significantly (*p* ≤ 0.05) different according to Turkey’s HSD.

It has been reported that engineered AgNPs suppress conidial germination and germ tube development, and significantly reduce conidial outgrowth, in a work by Jian et al. (Jian et al., 2021). These results suggest that AgNPs can effectively prevent phytopathogenic fungi from developing asexually. Regarding AgNP activity, research on various bacteria and fungi has shown that AgNP treatment can compromise cell membrane integrity and permeability. In addition, the RNA-Seq data showed that AgNPs according to the KEGG category can inhibit the transcription of genes associated with cellular energy expenditure and metabolism in *F. graminearum* (Jian et al., 2021). Similar results were recently observed in fungi, suggesting that the disruption of cellular energy expenditure and metabolic pathways is a key component of AgNPs’ antifungal efficacy (Table 4) (Shen et al., 2020).

**TABLE 4:** Previous study of mycelial growth inhibition activity of synthesized AgNPs.

Plants	Plant part	Zone of inhibition		References
		*F. oxysporum* (%)	*A. alternata* (%)	
*Buchanania lanzan, Abronia villosa*	leaves	47.08	65.86	(Abdelmoteleb et al., 2020), (Purohit et al., 2022)
*Salacia gambleana*	leaves	25	35	Nair et al. (2020)
*Mentha pulegium*	leaves	51	61	Rizwana and Alwhibi, (2021)
*Aaronsohnia factorovskyi*	leaves	85	77	Al-Otibi et al. (2021b)
*Malva parviflora*	leaves	80.7	83.0	Al-Otibi et al. (2021b)
*Plantago ovata*	leaves	81.48	86.67	This work

### Effect of AgNPs on the spore germination

Significant spore germination suppression of *F. oxysporum* and *A*. *alternata* were detected at a varied concentration of AgNPs, as shown in the results (Table 5). The highest quantities of biosynthesized AgNPs resulted in the greatest suppression of spore germination against *F. oxysporum* and *A*. *alternata* (Talie et al., 2020). For control, the germination rate was 100%. However, it reduced sharply with increased concentration of AgNPs. To be specific, 60.57 and 59.78% spore germination of *F. oxysporum* and *A*. *alternata* were inhibited at AgNO_3_ and the 25, 50, 75 and 100 μg/ml of biosynthesized AgNPs shown respectively, 56.39, 61.76, 67.35 and 76.30% of spore inhibition of *F*. *oxysporum* and 61.51, 65.31, 68.13 and 79.18% of spore inhibition of *A*. *alternata* and highest spore inhibition 76.30% (*F. oxysporum*) and 79.18% (*A*. *alternata*) were absorbed in 100 μg/ml concentration of biosynthesized AgNPs. Conidia germination is the key process for pathogen invasion, high inhibition rate at a low concentration of AgNPs against conidia germination could induce effective pathogen quantity and even avoid its infection of plants (Huang et al., 2022).

**TABLE 5:** Inhibition activity of AgNPs on spore germination of F. oxysporum and *A. alternata*

Treatment (%)	% Inhibition (Spore germination) F. oxysporum	% Inhibition (Spore germination) *A. alternata*
Control	0.00 ± 0.00c	0.00 ± 0.00c
AgNO_3_	60.57 ± 7.55ab	59.78 ± 1.83b
AgNPs		
25 μg/ml	56.39 ± 8.57b	61.51 ± 4.43b
50 μg/ml	61.76 ± 7.63ab	65.31 ± 7.09b
75 μg/ml	67.35 ± 4.75ab	68.13 ± 3.40ab
100 μg/ml	76.30 ± 5.84a	79.18 ± 3.75c

Values are means of three independent replicates (n = 3).±Indicate standard errors. Means followed by the same letter(s) within the same column are not significantly (*p* ≤ 0.05) different according to Turkey’s HSD.

## Conclusion

In this study, the highly stable silver nanoparticles were synthesized in an aqueous leaf extract medium by a simple, cheap and environmentally friendly biological strategy. The characterization of AgNPs was analyzed by UV-Vis result and shows that the peak at 414 nm confirmed the synthesis of AgNPs. The components such as aromatic compound (C=C), ether group (C-O), hydroxyl (-OH), and carboxyl (C-OH) and other biomolecules may have reduced and stabilized the silver ions to Ag NPs, confirmed by FTIR and Raman spectra. The crystalline nature of NPs has been demonstrated by XRD and SAED patterns. SEM and TEM analysis reveal spherical NPs with a particle size of 10–26 nm and an average size of biosynthesized AgNPs of 12.67 nm. The biosynthesized AgNPs show excellent catalytic properties in the degradation of MB and CR dyes under optimal experimental conditions. According to these results, the bio-reduction methods and biosynthesized NPs create new opportunities for the development of novel catalysts with efficient catalytic properties, recyclability, and stability. Furthermore, the synthesized AgNPs were found to have high antifungal activity even at low concentrations, especially in the case of *A. alternata*, since synthesized AgNPs are small and have better fungicidal activity.

## Data Availability

The data analyzed in this study is subject to the following licenses/restrictions: Data are available on the request to authors and journal. Requests to access these datasets should be directed to shaniraj1992@gmail.com.
