# Supplementary figures and images for: Phyto-fabrication of silver nanoparticles and their catalytic dye degradation and antifungal efficacy
Source: Front Chem. 2022 Sep 26;10:994721. doi: 10.3389/fchem.2022.994721 (PMC9548708; doi:10.3389/fchem.2022.994721)

Supplementary file

**Figure 5 DLS analysis**


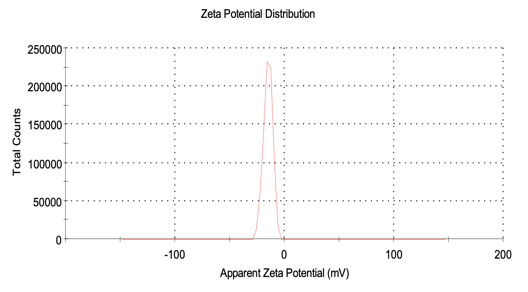

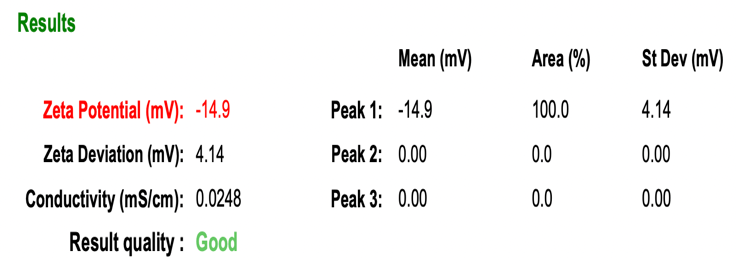


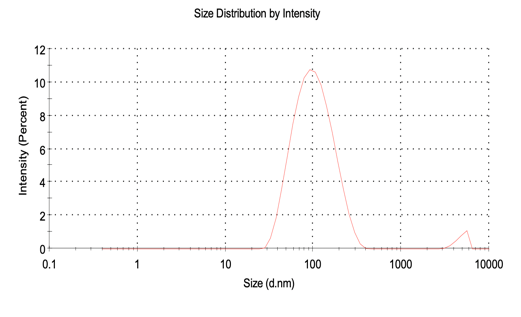

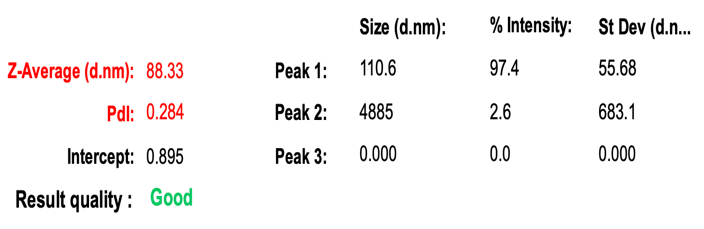


**
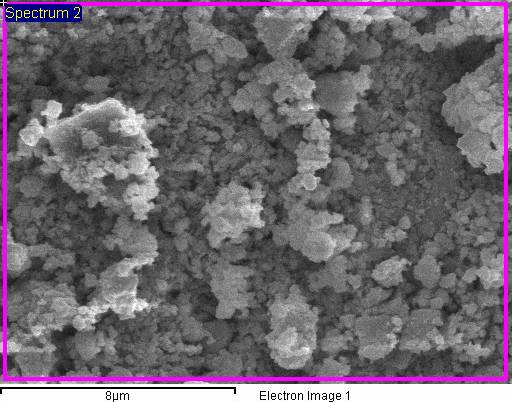

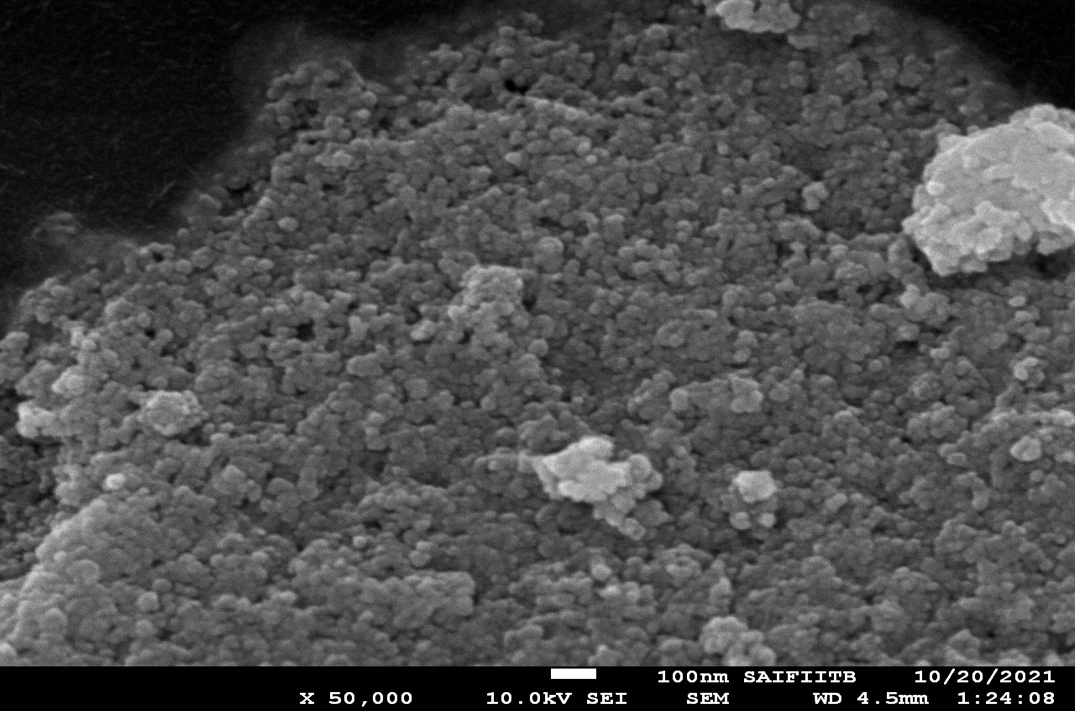
Figure 8 SEM analysis**

Supplement: Supplementary file 1 [file DataSheet1.docx]
